# Inhibition enhances capacity of sequence replay: a mean field model

**DOI:** 10.1186/1471-2202-12-S1-P195

**Published:** 2011-07-18

**Authors:** Álvaro Tejero-Cantero, Axel Kammerer, Christian Leibold

**Affiliations:** 1 Graduate School of Systemic Neurosciences, 82152 Martinsried, Germany; 2 Division of Neurobiology, Department of Biology II, LMU Munich, 82152 Martinsried, Germany

## 

**Figure 1:**
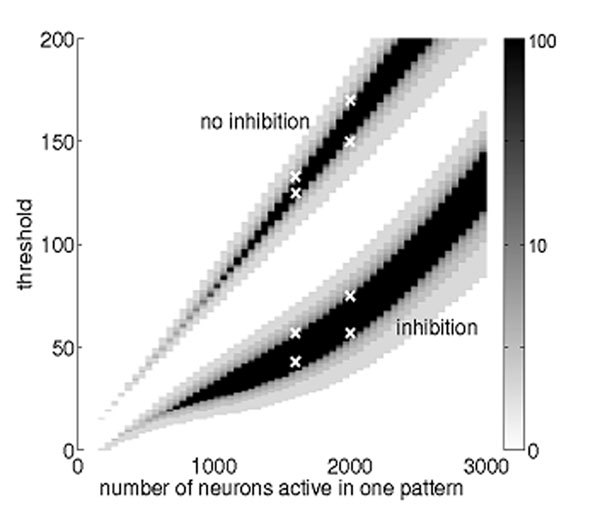
Regions of stable replay predicted by the mean field model without inhibition (upper wedge) and with inhibition (lower wedge) for a network of 10^5^ neurons. The gray level shows the number of iterations before replay fails as a function of the number of active neurons in a memory pattern and their common threshold. Black areas show stable replay over all 100 iterations. Inhibition allows stable replay of sparser patterns, thus increasing the capacity. White crosses mark the onset of stability in simulations of spiking neurons.

Sequences of neuronal activity patterns can be stored in networks of binary neurons with binary synapses. We investigate different forms of inhibition and their effect on such sequence memory, extending on a mean field approach [1]. There it was shown that successful replay requires a minimum degree of coding sparseness and that the capacity of the network increases as the code becomes sparser (Fig. 1). Here, we find that the introduction of global inhibition feedback makes sequence replay possible with an even sparser code, thereby increasing the memory capacity of the network (Fig. 1). At the same time, the range of firing thresholds compatible with replay becomes broader, suggesting a more robust behavior with noisy, biological neurons.

We further analyzed the effect of nonoptimal replay conditions: The replay performance degrades gracefully with the network exhibiting transient memory replay before falling into a state of silence or non memory-related activity. The regions of stable replay calculated from the mean field model were verified in cellular simulations.
